# Renewed call for action: Collection on gender inequality

**DOI:** 10.1016/j.eclinm.2022.101775

**Published:** 2022-11-29

In May 2019, *The Lancet* published the paper series Gender Equality, Norms, and Health highlighting research on gender inequalities in health, and called for health systems, programmes, and policies to transform gender norms and inequalities for a gender equitable society. The series recognised the systemic neglect to address gender inequalities which undermines the health of everyone. Following from this, in March 2020, *eClinicalMedicine* released the Gender Equality and Health collection in the continued commitment to fight gender disparities and inequality. The papers in this collection illustrate the critical importance of recognising gender in health and in the development of equal societies across the world. Discrimination against gender ultimately harms all parts of society and thus harms all of us and our health. These collections were curated in the hope of advancing gender equality worldwide.

Here in this issue, *eClinicalMedicine* highlights an update of the Gender Equality and Health collection to renew the call for action for a gender equitable society especially in the aftermath of the ongoing COVID-19 pandemic, which exacerbated the unequal value and treatment of women worldwide.

The collection includes papers explicitly looking at the effect of the COVID-19 pandemic on mental health, reproductive and maternal health services, and an increased vulnerability for women to household violence. Here specifically, research showed that women in urban Kenya had decreased access to menstrual supplies during the COVID-19 pandemic due to supply issues but also due to overall financial constraints. Along the same line, research in the collection found that women in rural India were seeking less health care for themselves or their children due to strains on the health system capacity and greater increases in household burdens during the pandemic. When focussing on migrant women compared with migrant men in India, women were more likely to stay unemployed during the pandemic and faced greater economic and food insecurities. Furthermore, research from northern Nigeria showed how the pandemic re-enforced gender norms, by female health-care workers taking on the increased domestic labor responsibilities during the pandemic, whereas male health-care workers increased their work shifts to 7 days a week to provide care.

Following on from the call for advancing women in science as highlighted in the previous collection, here we include three pieces focussing on gendered issues related to women leadership in academia and medicine. These papers demonstrate the barriers women are facing in their professional development. Especially, the collection highlights the detrimental effect of sexual harassment and provides clear recommendations how to combat this long-standing problem in academia. Furthermore, the collection includes a framework for overcoming systemic barriers to support women in advancing their careers in medicine and academia.

Societal burden of gender inequality and gender-based discrimination or violence negatively affects the health of women and this further can have detrimental effect on their children. Studies in this collection show that maternal gender discrimination perceived during pregnancy affects the mental health of the mother's children. Furthermore, partner-related support for postpartum women enhanced maternal and newborn health continuum of care as shown in a representative cohort study of Ethiopian women 5–9 weeks postpartum.

From this collection, we can learn that research is still needed to further show the interaction of gender-based discrimination related to the effect on women's health and lives but also the effect this can have on children and society. In the call for evidence-based research, gendered studies rely on quality data to provide support for policy changes and programme implementations. There is still a strong need for improvement to fill the data gap, as outlined in the collection. To provide meaningful change, the study calls for unbiased data collection which can in the future help to improve health outcomes for individuals of all gender.

Overall, looking back at 2019, the situation has not improved, and the mission is still unchanged: we must fight for equal rights independent of gender and end gender discrimination and bias. In the end, this will benefit us all.

**Explore the content in the collection below:**

Podcast: https://www.buzzsprout.com/1845316/11742053

Gender and the COVID-19 Pandemic: Multinational research indicates that we must support and compensate LMIC women's leadership in crises.: https://www.thelancet.com/journals/eclinm/article/PIIS2589-5370(22)00477-1/fulltext

COVID-19 burden, author affiliation and women's well-being: A bibliometric analysis of COVID-19 related publications including focus on low- and middle-income countries: https://www.thelancet.com/journals/eclinm/article/PIIS2589-5370(22)00336-4/fulltext

Product-access challenges to menstrual health throughout the COVID-19 pandemic among a cohort of adolescent girls and young women in Nairobi, Kenya: https://www.thelancet.com/journals/eclinm/article/PIIS2589-5370(22)00212-7/fulltext

Factors affecting delayed and non-receipt of healthcare during the COVID-19 pandemic for women in rural Maharashtra, India: Evidence from a cross-sectional study: https://www.thelancet.com/journals/eclinm/article/PIIS2589-5370(22)00470-9/fulltext

Strengthening health systems in crisis due to COVID-19 requires ending violence against female healthcare workers: https://www.thelancet.com/journals/eclinm/article/PIIS2589-5370(22)00248-6/fulltext

Gendered time use during COVID-19 among adolescents and young adults in Nairobi, Kenya: https://www.thelancet.com/journals/eclinm/article/PIIS2589-5370(22)00209-7/fulltext

COVID-19 and the gendered impacts on adolescent wellbeing: Evidence from a cross-sectional study of locally adapted measures in Ethiopia, Jordan, and Palestine: https://www.thelancet.com/journals/eclinm/article/PIIS2589-5370(22)00316-9/fulltext

Indian female migrants face greater barriers to post-Covid recovery than males: Evidence from a panel study: https://www.thelancet.com/journals/eclinm/article/PIIS2589-5370(22)00361-3/fulltext

Gender differences in work attendance among health care workers in Northern Nigeria during the COVID-19 pandemic: https://www.thelancet.com/journals/eclinm/article/PIIS2589-5370(22)00335-2/fulltext

Spousal Support and Work Performance during COVID-19 among Elected Women Representatives in Rural Bihar, India: https://www.thelancet.com/journals/eclinm/article/PIIS2589-5370(22)00472-2/fulltext

Networking practices and gender inequities in academic medicine: Women's and men's perspectives: https://www.thelancet.com/journals/eclinm/article/PIIS2589-5370(22)00068-2/fulltext

Harassment as a consequence and cause of inequality in academia: A narrative review: https://www.thelancet.com/journals/eclinm/article/PIIS2589-5370(22)00216-4/fulltext

Factors that influence the implementation of organisational interventions for advancing women in healthcare leadership: A meta-ethnographic study: https://www.thelancet.com/journals/eclinm/article/PIIS2589-5370(22)00244-9/fulltext

Maternal gender discrimination and child emotional and behavioural problems: A population-based, longitudinal cohort study in the Czech Republic: https://www.thelancet.com/journals/eclinm/article/PIIS2589-5370(22)00357-1/fulltext

The impact of partner autonomy constraints on women's health-seeking across the maternal and newborn continuum of care: https://www.thelancet.com/journals/eclinm/article/PIIS2589-5370(22)00445-X/fulltext

Effect of imbalanced sampling and missing data on associations between gender norms and risk of adolescent HIV: https://www.thelancet.com/journals/eclinm/article/PIIS2589-5370(22)00243-7/fulltext

